# Gut microbiota dynamics in SAMP8 mice: insights from machine learning and longitudinal analysis

**DOI:** 10.1128/spectrum.00635-25

**Published:** 2025-09-23

**Authors:** Yilang Ke, Aiping Zeng, Dang Li

**Affiliations:** 1 Fujian Key Laboratory of Vascular Aging, Department of Geriatrics, Fujian Institute of Geriatrics, Fujian Clinical Research Center for Senile Vascular Aging and Brain Aging, Fujian Medical University Union Hospital, Fuzhou, Fujian, China; North Carolina State University, Raleigh, North Carolina, USA

**Keywords:** gut microbiota, aging, SAMP8 mice, longitudinal study, machine learning

## Abstract

The gut microbiota plays a crucial role in maintaining host health, and its composition is significantly influenced by aging. The SAMP8 mouse model, known for its accelerated aging process, is widely used to study age-related changes. However, comprehensive longitudinal studies on gut microbiota dynamics in SAMP8 mice remain limited. We analyzed microbiota profiles of SAMP8 mice at 1, 3, 7, and 10 months (*n* = 6) using 16S rRNA sequencing. Alpha diversity (Shannon index) decreased significantly with age, while beta diversity revealed distinct clustering between young (1 and 3 months) and aged (7 and 10 months) SAMP8 mice. *Firmicutes*, *Actinobacteria*, and *Deferribacteres* declined significantly with age, whereas *Proteobacteria* and *Bacteroidetes* increased. At the genus level, *Allobaculum* and *unclassified_f_Lachnospiraceae* decreased significantly, whereas *Ruminiclostridium_5* and *Akkermansia* increased significantly in older mice. Microbiota trajectory analysis identified four aging-related patterns. For biomarker discovery, the young (1 and 3 months, *n* = 12) and aged (7 and 10 months, *n* = 12) groups were compared using Random Forest analysis, which identified 11 key taxa, with *Peptococcus* exhibiting the highest diagnostic accuracy (area under the curve = 0.78). These findings highlight the dynamic microbiota shifts during aging and identify *Peptococcus* as a potential biomarker for aging, offering insights into microbiota-aging interactions and potential translational targets.

**IMPORTANCE:**

Aging is associated with profound changes in microbial composition, yet the precise trajectories and key microbial signatures of aging remain incompletely understood. This study provides a comprehensive analysis of gut microbiota dynamics in aging SAMP8 mice. By identifying significant shifts in microbial diversity, composition, and aging-related trajectories, our findings highlight the progressive restructuring of gut microbiota with age. Understanding these changes is critical for uncovering potential microbial biomarkers of aging, which could serve as diagnostic tools or therapeutic targets to promote healthy aging. Notably, we demonstrate that some key taxa, such as *Peptococcus*, can differentiate young and aged microbiomes with high accuracy, offering insights into the potential role of gut microbiota in aging-related health decline. These findings provide a foundation for future research aimed at microbiota-targeted interventions, such as probiotics or dietary modifications, to mitigate age-associated diseases and improve lifespan and health span.

## INTRODUCTION

Aging is an inevitable biological process characterized by progressive functional decline across multiple systems, including the brain. One of the most profound age-related changes in the brain is cognitive decline, which can manifest as mild cognitive impairment or progress to severe neurodegenerative diseases such as Alzheimer’s disease (AD). AD is a progressive neurodegenerative disorder characterized by memory loss, cognitive dysfunction, and behavioral changes, ultimately leading to significant disability and mortality (1). The etiology of AD is complex, involving genetic, environmental, and lifestyle factors (2, 3). Recent studies have highlighted the gut microbiota as a critical modulator of aging (4) and AD (5) pathogenesis.

The gut microbiota has emerged as a significant factor influencing host health and disease, including aging and neurodegenerative processes. Comprising over 100 trillion symbiotic microorganisms (6), the gut microbiota resides in the host’s gastrointestinal tract and plays a crucial role in maintaining gut homeostasis, modulating immune responses, and influencing metabolic processes (7). Aging is associated with significant changes in the composition and diversity of the gut microbiota, characterized by a decline in microbial diversity and an increase in potentially pathogenic taxa (4). These age-related shifts have been linked to increased gut permeability, systemic inflammation, and cognitive decline. Notably, several studies have reported alterations in the gut microbiota of Alzheimer’s disease patients (8–10), suggesting a potential gut-brain axis mechanism underlying neurodegenerative processes.

The Senescence-Accelerated Mouse Prone 8 (SAMP8) mouse model (11, 12) is widely recognized for its suitability in studying aging-related deficits in learning and memory. Research conducted by our team (13) and others (14) has previously shown that SAMP8 mice exhibit significant cognitive declines as they age. Recent studies have demonstrated that microbiota-targeted interventions (15, 16) and fecal microbiota transplantation (17) can alleviate age-associated deficits in cognition and muscle function. Previous research has primarily focused on characterizing the gut microbiota changes in SAMP8 and Senescence-Accelerated Mouse Resistant 1 (SAMR1) at individual time points (18, 19). While a recent study has begun to explore longitudinal changes across different ages (17), comprehensive longitudinal studies integrating machine learning remain scarce.

In parallel, machine learning approaches are emerging as powerful tools for identifying microbiota signatures linked to aging. For example, gut microbiome-based aging clocks have achieved age-prediction accuracies within 5–10 years in adults (20), while gut microbiota combined with urine metabolomics has been used to predict chronological age (21). Machine learning models have also distinguished cognitively exceptional older individuals (“Superagers”) from typical agers based on gut microbiome features (22). These advances underscore the value of computational methods, such as Random Forest, for robust biomarker discovery in microbiome aging research.

Therefore, this study aimed to (i) characterize longitudinal changes in gut microbiota composition and diversity in SAMP8 mice at four different ages: 1, 3, 7, and 10 months, (ii) identify aging-related trajectories and key taxa via time-series clustering and linear regression, and (iii) use Random Forest to pinpoint microbial biomarkers distinguishing young and aged mice. The novelty lies in combining a longitudinal design with advanced computational analyses to provide statistically supported evidence for specific taxa as potential biomarkers of age, offering new insights into microbiota-aging interactions and potential translational targets.

## MATERIALS AND METHODS

### Animals

Twenty-four male SAMP8 mice were sourced from the Huafukang Company in Beijing, China. The mice were housed in a specific pathogen-free environment and provided with free access to a standard laboratory mouse diet and water *ad libitum*. The mice were divided into four age-matched cohorts, each comprising six mice: 1-, 3-, 7-, and 10-month-old groups. This study was conducted in strict accordance with the guidelines established by the National Institutes of Health (NIH) and the Fujian Regulations on Laboratory Animal Management.

### Fecal sample collection

To ensure the consistency and integrity of our experimental data, fecal samples from all mice were collected simultaneously. Prior to collection, each mouse was individually housed in a cage that had been separately sterilized by autoclaving to eliminate the risk of cross-contamination. We carefully collected three to four fecal pellets from each mouse and transferred them into a sterile 1.5 mL microcentrifuge tube. The samples were immediately submerged in liquid nitrogen to arrest any biochemical activity. Within 2 hours of collection, the samples were transported to a −80°C freezer for long-term storage, thereby ensuring optimal preservation of the fecal microbiota.

### DNA extraction and 16S rRNA sequencing

Fecal bacterial genomic DNA was extracted from all stool samples using the QIAamp DNA Stool Mini Kit (Qiagen, catalog number 51504) according to the manufacturer’s instructions and stored at −80°C. DNA concentration and purity were measured using a NanoDrop 2000 spectrophotometer (Thermo Fisher Scientific, USA), and integrity was assessed by 1% agarose gel electrophoresis. Samples were required to have an A260/280 ratio between 1.8 and 2.0, an A260/230 ratio ≥ 2.0, and a DNA concentration ≥ 20 ng/µL to proceed to library preparation and sequencing. An appropriate volume of the sample was transferred to a centrifuge tube and diluted to 1 ng/µL. The diluted genomic DNA was then used as the template for amplifying the V4 region of the 16S rRNA gene using primers 515F (GTG CCA GCM GCC GCG GTAA) and 806R (GGA CTA CHV GGG TWT CTAAT). The PCR reaction system consisted of 15 µL Phusion Master Mix (2×), 3 µL Primer (2 µM), 10 ng of template DNA, and ddH_2_O to a final volume of 30 µL. PCR cycling involved an initial denaturation at 98°C for 1 minute, 35 cycles of denaturation, annealing at 50°C, extension at 72°C, and a final extension at 72°C for 5 minutes, ending at 4°C.

To minimize batch effects, all fecal samples were stored at −80°C immediately after collection and processed using the same DNA extraction kit and protocol. Samples from different age groups were extracted and sequenced together, where possible, to ensure consistency.

### Illumina sequencing

For the preparation of the library, the TruSeq DNA PCR-Free Sample Preparation Kit by Illumina in San Diego, USA, was employed. The library’s quantification was achieved through the employment of both the Qubit and Q-PCR techniques. After successful quantification of the library, sequencing was conducted on the Illumina HiSeq2500 PE250 platform.

### Data processing

Residual batch effects from sequencing runs were assessed and corrected using the ComBat function from the “SVA” R package, with sequencing batch as a covariate. The initial segregation of sample data was conducted based on barcode sequences and PCR-amplified primer sequences, separating individual sample reads from the overall dismounting data. The integration and preliminary filtering of these sequences were accomplished using FLASH software (version 1.2.7) (23), which facilitated the splicing of reads following sequence trimming. Further refinement involved the use of QIIme software (version 1.7.0) (24), which allowed for the precise interception and length-based filtration of tags. The extraction of effective tags was then performed, leveraging the UCHIME Algorithm (25) to match sequences against the gold database and to eliminate any chimeric sequences identified.

Following this, the Uparse software (version 7.0.1001) (26) was utilized to cluster these effective tags from all collected samples into operational taxonomic units (OTUs), with a default similarity threshold of 97%. Species annotation was then conducted on the representative sequences from these OTUs. For detailed taxonomic classification, the species annotation process was executed using the Mothur analytical method, applied to the SSUrRNA (27) database of SILVA (28), with a specified similarity threshold range set between 0.8 and 1.0 to ensure rigorous taxonomic identification.

### Statistical analysis

The alpha diversity indices and the beta diversity index (principal coordinate analysis [PCoA] and non-metric multidimensional scaling [NMDS]) of gut microbiota were performed using the R packages vegan (version 2.6-8) (https://github.com/vegandevs/vegan) and visualized using ggplot2 (version 3.5.1) (29). The gut microbiota composition was processed and analyzed using the R packages “dplyr” (version 1.1.4) (30) and visualized using the “aplot” package (version 0.2.4) (31). To assess the variance between multiple groups, the Kruskal-Wallis test was deployed, supplemented by subsequent analysis using a Bonferroni *post hoc* test to elucidate group-specific differences. Time-series data of gut microbiota were analyzed using the ClusterGVis package (version 0.1.2) (32). The data were normalized and clustered using the clusterData function with the Mfuzz (version 2.66.0) (33, 34). The correlation between genera and age was analyzed by linear regression using the “stats” package (version 4.4.2).

To identify differentially abundant microbial taxa among groups, we performed linear discriminant analysis effect size (LEfSe) analysis using the microeco R package (version 1.12.0) (35). To further explore the predictive power of the identified taxa, we selected microbial genera with *P* < 0.05 from the LEfSe analysis as features for machine learning. These genera were used to train classification models to assess their ability to predict sample groupings accurately. Feature selection and importance ranking in the data set were executed through the Random Forest algorithm due to its efficacy in managing high-dimensional data, setting the number of trees at 100, and utilizing the Gini index for feature importance evaluation. To verify the robustness of the model, cross-validation was conducted using a fivefold approach, with accuracy serving as the principal measure for assessing model performance. Feature importance scores were determined, and cumulative importance was analyzed to pinpoint the most critical features. These computational tasks were performed using the R “randomForest” package (version 4.7-1.2) (36) and “caret” package (version 7.0-1) (37). The prediction performance of the model was evaluated by the area under the receiver operating characteristic (ROC) curve using the “pROC” package (version 1.18.5) (38).

## RESULTS

### Aging-related changes in microbial diversity

**Fig 1:**
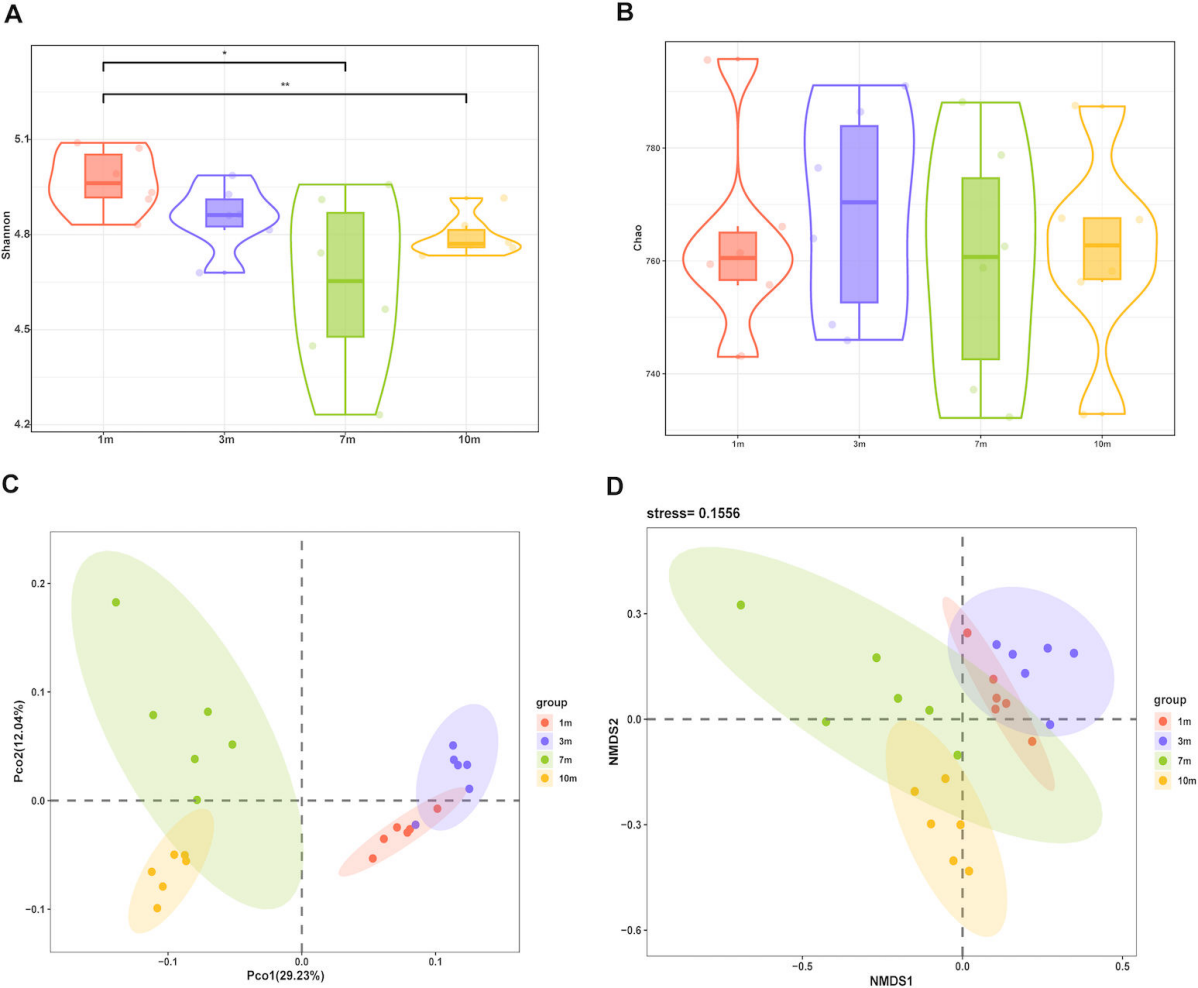
Microbial diversity in SAMP8 during aging. (**A**) Shannon diversity index of different groups. (**B**) Chao diversity index of different groups. (**C**) Beta diversity of the genera analyzed by PCoA. (**D**) Beta diversity of the genera analyzed by NMDS. *n* = 6.

To understand how aging affects gut microbiota diversity in SAMP8 mice, we evaluated microbial alpha diversity across different age groups using the Shannon and Chao diversity indices (Fig. 1A). Compared to 1-month-old SAMP8 mice, both 7- and 10-month-old mice exhibited a significant decline in the Shannon index, indicating reduced microbial diversity. However, no significant age-related changes were observed in the Chao index, which measures species richness. This indicates that aging in SAMP8 mice is associated with reduced diversity of the gut microbiota without a major loss of species richness. Such reductions in diversity may compromise the stability and resilience of the gut ecosystem, increasing susceptibility to dysbiosis and functional declines in aged mice.

To further elucidate the distributional differences among these groups, we used weighted UniFrac PCoA and NMDS to assess β-diversity and visualize the differences between age groups (Fig. 1B). These analyses revealed distinct clustering patterns, with four major clusters emerging across all samples. Specifically, the 1- and 3-month-old groups clustered closely together, while the 7- and 10-month-old groups formed a separate cluster. This pattern suggests a clear separation in gut microbiota composition between younger (1- and 3-month-old) and older (7- and 10-month-old) SAMP8 mice, indicating significant age-related shifts in microbial community structure.

### Aging-related alterations in microbiota composition

To elucidate the longitudinal changes in gut microbiota composition during the aging process, we analyzed the relative abundance of bacterial phyla and genera in SAMP8 mice across four distinct age groups: 1, 3, 7, and 10 months.

**Fig 2:**
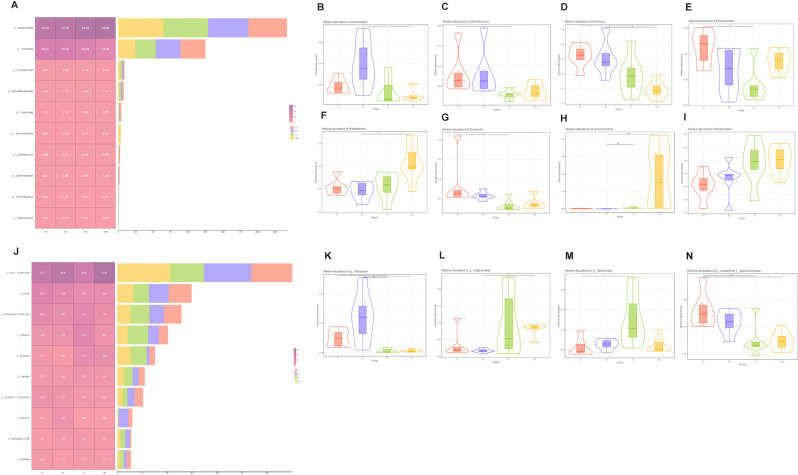
Compositional differences in SAMP8 mice during aging. Heatmap and stacked bar chart illustrating the relative abundances and distribution patterns of the top 10 predominant microbial phyla (**A**) and genera (**J**) across different age groups. The heatmap displays the relative abundance of each phylum or genus within each age group, with the color scale ranging from low abundance (magenta) to high abundance (dark violet). The numbers within each cell represent the exact relative abundance values (expressed as a percentage). The stacked bar chart provides a visual summary of these abundances, with each color bar representing a group and different lengths indicating the relative contribution of each phylum or genus to the total microbial community. Panels B–I show the comparative analysis of relative abundances for significant phyla, including *Actinobacteria* (**B**), *Deferribacteres* (**C**), *Firmicutes* (**D**), *Patescibacteria* (**E**), *Proteobacteria* (**F**), *Tenericutes* (**G**), *Verrucomicrobia* (**H**), and *Bacteroidetes* (**I**). Panels K–N show the comparative analysis of relative abundances for significant genera, including *Allobaculum* (**K**), *Alloprevotella* (**L**), *Bacteroides* (M), and *unclassified_f_Lachnospiraceae* (**N**). Statistical analyses were performed using the Kruskal-Wallis test, followed by a Bonferroni *post hoc* test to assess group-specific differences. Symbols * and ** indicate statistical significance at *P <* 0.05 and *P <* 0.01*,* respectively.

Our results revealed significant alterations in microbial community structure over time. Specifically, *Bacteroidetes* and *Firmicutes* consistently dominated the gut microbiota across all stages. In contrast, *Verrucomicrobia*, which was almost undetectable in younger mice, increased notably in 10-month-old SAMP8 mice (Fig. 2A). As detailed in Table S1, significant differences in abundance were observed among the age groups for eight bacterial phyla, except for *Epsilonbacteraeota* and *Cyanobacteria*. Notably, the relative abundance of *Firmicutes*, *Actinobacteria*, *Deferribacteres*, and *Tenericutes* decreased with age, while *Proteobacteria* and *Bacteroidetes* increased (Fig. 2B through I). Consistent with these phylum-level shifts, we calculated the *Firmicutes*-to-*Bacteroidetes* (F/B) ratio (log₂-transformed) across age groups. Kruskal-Wallis analysis revealed significant overall differences in the F/B ratio among the four age groups (χ² = 11.91, df = 3, *P* = 0.00771; Table S2). *Post hoc* pairwise comparisons (Bonferroni-adjusted) showed that the ratio was significantly lower in 10-month-old SAMP8 mice compared with 1-month-old mice (adjusted *P* = 0.013), while other group comparisons were not statistically significant.

Additionally, we examined the top 10 most abundant bacterial genera (Fig. 2J). Among these, significant differences in abundance were observed for the genera *Allobaculum*, *Alloprevotella*, *Bacteroides*, and *unclassified_f_Lachnospiraceae* across the four age groups (see Table S3). Specifically, the relative abundance of *Allobaculum* and *unclassified_f_Lachnospiraceae* decreased with age (Fig. 2K through N).

These findings highlight the dynamic nature of gut microbiota composition in SAMP8 mice as they age, with distinct patterns emerging at different life stages, suggesting age-related shifts in microbial community structure.

### Aging-related microbiota trajectories

To investigate the age-related trajectories of gut microbiota, 128 genera were classified into four clusters based on their distribution across different age groups. The four clusters are as follows: (i) genera that continuously decreased from 1 to 7 months of age, then increased at 10 months, but remained lower than at 1 month (*n* = 37); (ii) genera that increased from 1 to 10 months of age (*n* = 26); (iii) genera that increased from 1 to 7 months of age and recovered at 10 months (*n* = 27); and (iv) genera that increased from 1 to 3 months of age, then continuously decreased until 10 months (*n* = 38). Detailed information on the gut microbiota of each cluster is provided in Table S4.

**Fig 3:**
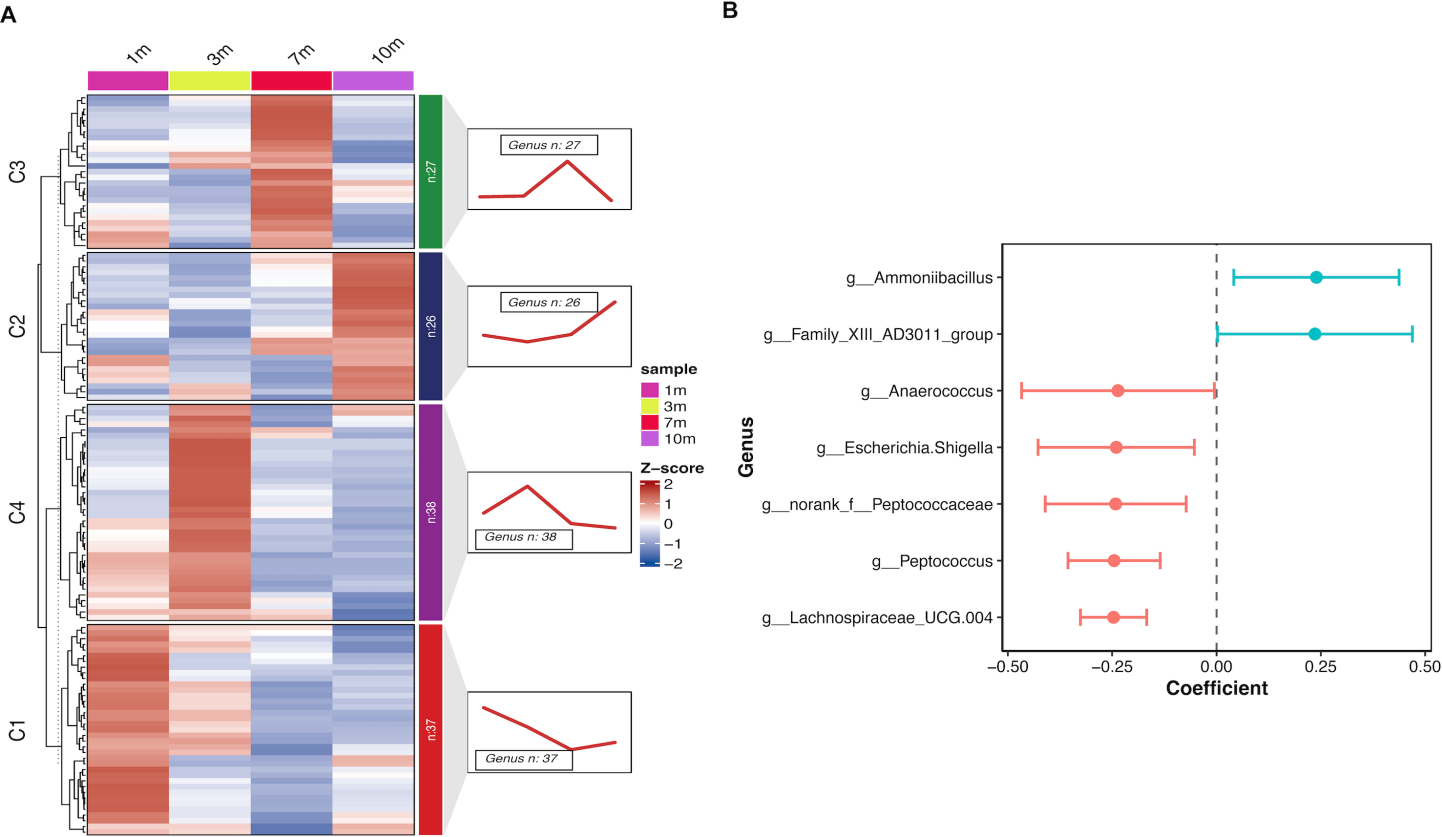
Microbiota distribution trajectories over the aging process. (**A**) Heatmap (left panel) showing genera classified into different clusters (C1: cluster 1, C2: cluster 2, C3: cluster 3, and C4: cluster 4) based on their distribution patterns across different age groups (1m: 1 month old, 3m: 3 months old, 7m: 7 months old, and 10m: 10 months old). The right panel displays the longitudinal trajectories of the four clusters, illustrating how the relative abundance of each cluster changes over time. (**B**) Horizontal bar plot showing the standardized coefficients for significant genera and age points, analyzed using linear regression. Each bar represents the strength and direction of the association between the genus abundance and age.

To verify the correlation between gut microbiota and age, a linear regression model was employed (see Table S5). As shown in Fig. 3B, seven genera exhibited significant changes with age. The relative abundance of *Anaerococcus* (cluster 1, *P* = 0.048), *Escherichia-Shigella* (cluster 1, *P* = 0.031), *norank_f_Peptococcaceae* (cluster 1, *P* = 0.025), *Peptococcus* (cluster 1, *P* = 0.011), and *Lachnospiraceae_UCG.004* (cluster 1, *P* = 0.006) decreased significantly with increasing age. In contrast, the relative abundance of *Ammoniibacillus* (cluster 4, *P* = 0.035) and *Family_XIII_AD3011_group* (cluster 4, *P* = 0.049) increased significantly with age.

### Aging-related signature of the specific microbiota

**Fig 4:**
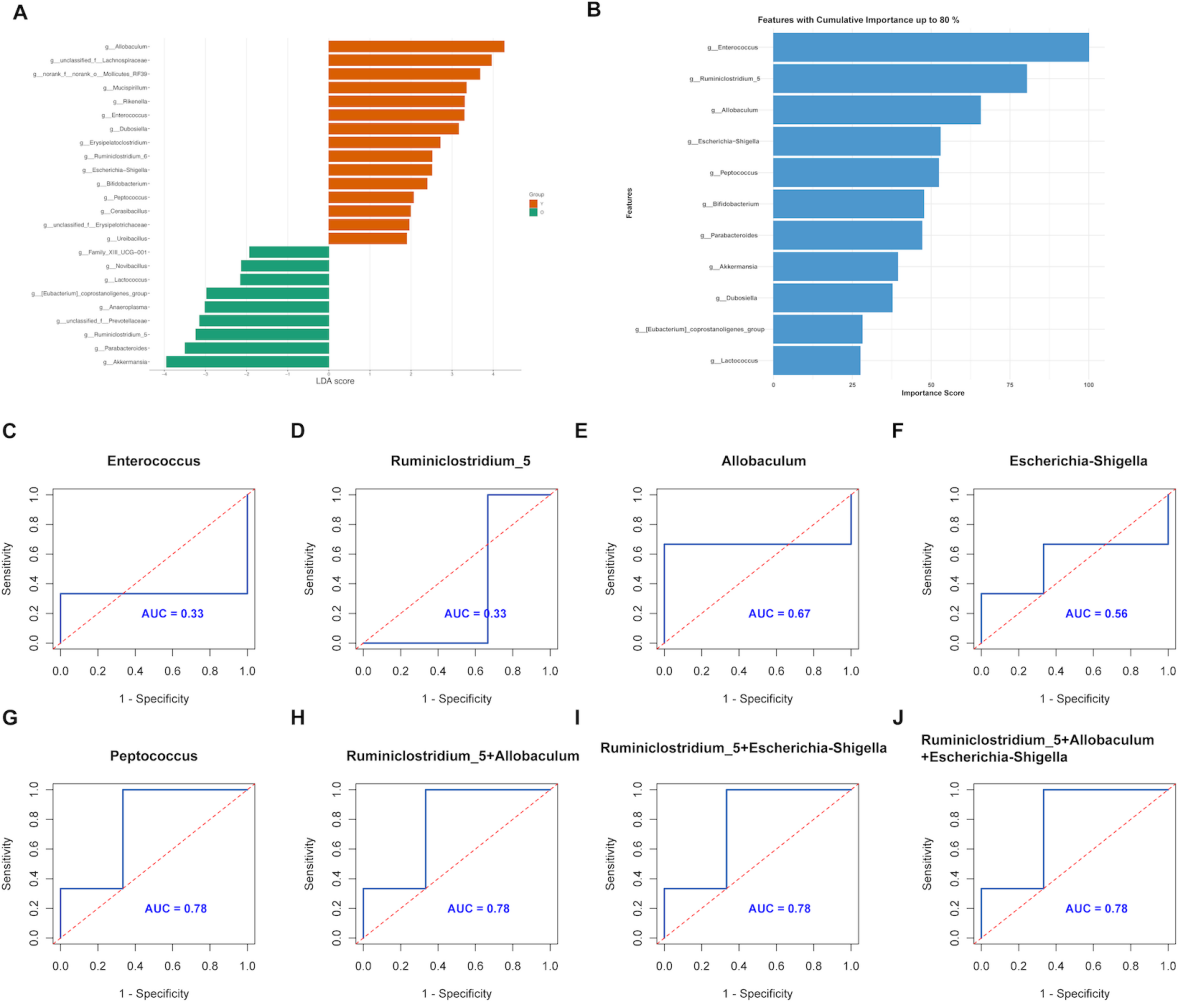
Identification of key aging-related genera in SAMP8 mice. (**A**) Signature genera distinguishing young (1- and 3-month-old) and aged (7- and 10-month-old mice) SAMP8 mice were identified using linear discriminant analysis (LDA). (**B**) Random Forest analysis identified important aging-related genera in SAMP8 mice. The bar plot illustrates the features ranked by their importance score in descending order. The length of each bar represents the feature’s contribution to the model’s predictive power, with longer bars indicating higher importance. (**C–J**) ROC curve analysis in the validation cohort to evaluate the classification ability of individual genera and genus combinations in predicting different age groups. *Enterococcus* (**C**), *Ruminiclostridium_5* (**D**), *Allobaculum* (**E**), *Escherichia-Shigella* (**F**), *Peptococcus* (**G**), *Ruminiclostridium_5 + Allobaculum* (**H**), *Ruminiclostridium_5 + Escherichia-Shigella* (**I**), and *Ruminiclostridium_5 + Allobaculum + Escherichia-Shigella* (**J**).

Previous analyses have shown that 1- and 3-month-old SAMP8 mice exhibit similar gut microbiota compositions, whereas 7- and 10-month-old SAMP8 mice share comparable microbiota profiles. To further elucidate the gut microbiota signature of young and aged mice, we combined 1- and 3-month-old mice into a “young” group (Y) and 7- and 10-month-old mice into an “aged” group (O) for comparative analysis using the linear discriminant analysis effect size method. This analysis identified 15 genera, including *Allobaculum*, *Enterococcus, Peptococcus*, *Bifidobacterium*, and *Ureibacillus*, which were enriched in the young group, while 9 genera, including *Ruminiclostridium_5*, *Parabacteroides*, and *Akkermansia*, were enriched in the aged group (Fig. 4A; Table S6).

### Machine learning identification of key aging-related genera

To differentiate the gut microbiota between young and aged SAMP8 mice, we developed a machine learning model using the Random Forest algorithm with fivefold cross-validation. The genera used for model training were identified through LEfSe analysis. The Random Forest model achieved an average cross-validation accuracy of 79.00%, with additional performance metrics as follows: sensitivity (0.83), specificity (0.75), precision (0.77), recall (0.83), F1 score (0.80), and the area under the ROC curve (AUC = 0.81). By evaluating feature importance and setting a cumulative importance threshold that accounted for 80% of total feature importance (see Table S7), we identified 11 key genera that significantly contributed to distinguishing between young and aged groups. The top five aging-related taxa were *Enterococcus*, *Ruminiclostridium_5*, *Allobaculum*, *Escherichia-Shigella*, and *Peptococcus* (Fig. 4B).

We further evaluated the diagnostic efficacy of these top five individual gut microbiota taxa and their various combinations in distinguishing between young and aged states using receiver operating characteristic curve analysis. The areas under the curve for the top five individual taxa were 0.33, 0.33, 0.67, 0.56, and 0.78, respectively. Additionally, we explored the diagnostic efficacy of different combinations of these taxa, including pairs, triplets, quadruplets, and the full combination of all five taxa. The results showed that the combinations of *Ruminiclostridium_5* and *Allobaculum*, *Ruminiclostridium_5* and *Escherichia-Shigella*, and *Ruminiclostridium_5*, *Allobaculum*, and *Escherichia-Shigella* achieved an AUC of 0.78, comparable to the diagnostic efficacy of *Peptococcus* alone. Other combinations of taxa exhibited lower diagnostic efficacy, with AUC values not exceeding 0.78 (see Fig. S1). These findings indicate that *Peptococcus* alone provides relatively better diagnostic performance for distinguishing between young and aged states. This suggests that specific gut microbiota taxa, particularly *Peptococcus,* could serve as a potential biomarker for age-related changes in SAMP8 mice.

## DISCUSSION

In this study, we comprehensively delineated the chronological shifts in microbial diversity and composition across different age stages in SAMP8 mice. Our longitudinal analysis revealed four distinct microbiota trajectories associated with aging, highlighting the dynamic changes in gut microbiota throughout the aging process. Notably, we identified a candidate genus, *Peptococcus*, which can effectively distinguish between the young and old stages.

To our knowledge, this is one of the very few longitudinal studies to systematically analyze the dynamic changes in gut microbiota composition in SAMP8 mice throughout the aging process. Previous studies have primarily focused on comparing gut microbiota alterations between age-matched SAMP8 and SAMR1 mice. For instance, Zhan et al. (18) observed distinct gut microbiota compositions in 7-month-old SAMP8 mice compared with age-matched SAMR1 mice. Similarly, another study documented divergent gut microbiota patterns between the two strains at 8 months of age (19). A recent study observed longitudinal gut microbiota changes across aging and highlighted potential links to cognitive decline via short-chain fatty acids (SCFAs) and lipopolysaccharide-related pathways (17). However, that work primarily focused on metabolic pathway analysis and did not employ advanced computational approaches for biomarker identification. In contrast, our investigation examined gut microbiota profiles at four distinct ages (1, 3, 7, and 10 months) in SAMP8 mice, integrating both traditional statistical methods and advanced tools such as time-series clustering and Random Forest modeling. This combined approach enabled us to more comprehensively characterize age-related changes in gut microbial communities and identify specific taxa over the course of aging.

It is well established that aging reduces the diversity of the gut microbiota (39, 40). Alpha diversity, which serves as a key indicator of gut microbiota health, has been associated with various age-related conditions, including Alzheimer’s disease (41), hypertension (42), and diabetes (43). In our study, we observed a significant decrease in microbial alpha diversity (as indicated by the Shannon index) with age in SAMP8 mice. Conversely, the Chao index, which primarily reflects the number of operational taxonomic units, showed little change with age. This finding aligns with previous research (44), which similarly reported a decline in the Shannon index in older individuals without a corresponding change in the Chao index. The Shannon index is an assessment of microbial community diversity (45), while the Chao index estimates species richness (46). The observed discrepancy between these indices can be attributed to their distinct measurement focuses. Our results suggest that microbial diversity, rather than mere richness, may be a more critical factor in the age-related changes of the gut microbiota in SAMP8 mice.

The composition and balance of gut microbiota are highly dependent on age, often accompanied by significant shifts in microbial communities. Across all age groups, *Bacteroidetes* and *Firmicutes* were the two most dominant phyla. The ratio of *Firmicutes* to *Bacteroidetes* is considered a reliable marker of gut health and an index of aging, with imbalances linked to increased gut permeability and inflammation (47). In our study, aging SAMP8 mice exhibited a concurrent increase in *Bacteroidetes* abundance and a significant decrease in *Firmicutes*, leading to a pronounced reduction in the F/B ratio at 10 months of age (Table S2). Similar age-related reductions in the F/B ratio have been reported in elderly human populations (48, 49) and in aging mice (50), further supporting the use of this marker to indicate gut microbiota aging.

Another phylum of interest is *Verrucomicrobia*, which is characterized by anti-inflammatory properties and is important for gut health (51). Previous research by Bárcena et al. (52) reported a reduction in *Verrucomicrobia* in the aged skin of progeria mice (Hutchinson–Gilford progeria syndrome model mice), attributing this to microbial dysbiosis and destabilization of the gut community during early aging. Intriguingly, our study revealed that 10-month-old SAMP8 mice exhibited a significantly higher abundance of *Verrucomicrobia* compared to other age groups. Consistent with our findings, another study found an increased abundance of *Verrucomicrobia* in elderly individuals, with *Verrucomicrobia* positively associated with the inflammatory marker IFNγ (53). Thus, the role of *Verrucomicrobia* in the aging process remains unclear and requires further investigation.

The SAMP8 mouse model is widely recognized for its suitability in studying aging-related deficits in learning and memory. Previous research has demonstrated that SAMP8 mice exhibit declines in learning ability as early as 3 months of age (54, 55), with memory impairments becoming apparent by 6–7 months (56, 57) and progressing to severe deficits resembling late-stage Alzheimer’s disease by 10 months (58). In this study, we identified four longitudinal trajectories in the aging process of SAMP8 mice. Specifically, the abundance of genera in cluster 4, such as *Ammoniibacillus* and *Family_XIII_AD3011_group*, peaked at 3 months of age. Evidence from inflammatory and clinical cohorts indicates that *Family_XIII_AD3011_group* is associated with inflammatory markers or pathways in ulcerative colitis and psychiatric contexts, and Mendelian randomization further links this genus to CXCL11-mediated inflammatory signaling (59–61). Zhang et al. (62) found a strong association between *Family_XIII_AD3011_group* and IL-5. IL-5, a neuroprotective cytokine (63) produced by Th2 cells and ILC2s (64), has been shown to transiently improve spatial learning, potentially by modulating downstream neuroinflammatory processes rather than directly reducing Aβ burden in an AD mouse model (65). Human cortical data further show altered IL-5 levels in AD brains (66), while genetic epidemiology suggests a nominally protective association of circulating IL-5 with AD risk (67). Collectively, these observations raise a hypothesis that the transient peak of cluster-4 taxa (including *Family_XIII_AD3011_group*) at 3 months may be associated with immune signaling (e.g., IL-5) relevant to early learning decline in SAMP8. However, current evidence is associative and largely derived from non-SAMP8 context and should be interpreted cautiously pending functional validation.

Conversely, the abundance of genera in cluster 1, such as *Erysipelatoclostridium*, was lowest at 7 months of age. Previous studies have shown that higher levels of *Erysipelatoclostridium* are associated with increased SCFA concentrations in the gut (68, 69). SCFAs are crucial for maintaining the integrity and function of the gut barrier (70). Chen et al. (71) reported that supplementation with probiotics (specifically *Lactobacillus casei* Shirota) could increase the abundance of beneficial bacteria such as *Erysipelatoclostridium*, *Mucispirillum*, and *Anaerotruncus*, as well as the level of short-chain fatty acids, thereby attenuating age-related sarcopenia in SAMP8 mice. Xiao-Hang et al. (72) demonstrated that multi-strain probiotic treatment altered gut microbiota composition, resulting in increased abundance of *Erysipelatoclostridium* and improved cognitive function in aging SAMP8 mice. Recent studies have highlighted the association between altered gut microbiota, specifically those producing SCFAs, and the pathogenesis of Alzheimer’s disease (73). Taken together, these results suggest that the memory impairments observed in 6- to 7-month-old SAMP8 mice may be associated with the decline in *Erysipelatoclostridium*.

Based on our findings, the genus *Peptococcus* exhibits significant diagnostic efficacy in distinguishing young and old SAMP8 mice, with higher abundance observed in young SAMP8 mice. As a member of the Firmicutes phylum, *Peptococcus* has been shown to play a crucial role in antioxidant processes, as evidenced by its negative correlation with malondialdehyde levels (74). Additionally, *Peptococcus* exhibits a protective effect against inflammatory diseases (75, 76). Oxidative stress and inflammation are key drivers in the pathogenesis and progression of AD, creating a vicious cycle that exacerbates neurodegeneration (77). Given that *Peptococcus* has been shown to mitigate oxidative stress and inflammation, it is plausible that this genus may influence the development of AD-like pathology in SAMP8 mice. Specifically, the enrichment of *Peptococcus* in young SAMP8 mice could potentially counteract the oxidative stress and inflammation associated with aging and neurodegeneration, thereby delaying the onset or progression of AD-like symptoms.

Our findings have several translational implications for microbiota-targeted therapy in aging. The observed decline in alpha diversity and the age-related loss of SCFA-producing taxa suggest two complementary intervention avenues: (i) enhancing SCFA production through dietary fiber, prebiotics, or probiotic consortia that promote beneficial taxa, and (ii) reducing the expansion of potentially pro-inflammatory bacterial groups such as *Proteobacteria* to restore microbial balance. Previous studies in SAMP8 mice have shown that probiotic supplementation can ameliorate age-related cognitive and physical decline, supporting the feasibility of such strategies in this model. Fecal microbiota transplantation has also been demonstrated to remodel gut communities and improve aging-related outcomes in murine models, although its clinical translation requires careful safety considerations. Notably, our Random Forest model identified *Peptococcus* as the top-ranking genus for discriminating between young and aged mice. This finding supports *Peptococcus* as a candidate biomarker for early detection of accelerated microbiota aging, monitoring therapeutic responses, and potentially guiding targeted microbiome restoration strategies. Together, these insights provide a mechanistic and testable pathway for translating microbiome-based findings into interventions aimed at mitigating age-related gut dysbiosis and its systemic consequences.

This study has several limitations that need to be acknowledged. First, the sample size was relatively small (*n* = 6 per age group; *n* = 12 per class in machine learning analyses), which may limit the statistical power, especially in machine learning applications, and reduce the broader applicability of our findings. Second, only male mice were used to reduce variability from hormonal cycles; however, this limits the applicability of the results across sexes, and future studies should include both male and female mice to assess sex-specific microbiota-aging interactions. Additionally, our results are based solely on observational data and have not been confirmed through experimental approaches. This means that while we can identify associations, we cannot definitively prove cause-and-effect relationships. Furthermore, the lack of functional analyses makes it difficult to translate our findings into practical recommendations or actionable insights.

Future research should aim to address these limitations by increasing sample sizes, including both sexes, and incorporating experimental validation to support our observations. Including functional studies could also help elucidate the underlying mechanisms more clearly. These improvements would strengthen the reliability of the findings and enhance their potential use in developing strategies for disease prevention and treatment.

### Conclusions

In summary, our longitudinal study provides novel insights into the dynamic changes in gut microbiota during aging and their potential impact on cognitive decline. The findings highlight the importance of microbial diversity and specific taxa like *Peptococcus* in modulating age-related neurodegenerative processes. To move toward translation, functional validation is needed to establish the causal role of *Peptococcus,* for example, through targeted supplementation or depletion of *Peptococcus* to establish causal links. Additionally, broader animal and human studies are required to confirm generalizability. Such efforts will be critical for developing microbiota-targeted interventions to mitigate age-related cognitive decline.

## Data Availability

The raw sequencing data are available in the NCBI Sequence Read Archive (SRA) under the study accession number SRP606317, within BioProject PRJNA1301221.

## SUPPLEMENTAL MATERIAL

Fig. S1 spectrum.00635-25-s0001.tif ROC curve analysis for various combinations of the top five genera identified by the Random Forest model.

Supplemental tables spectrum.00635-25-s0002.docx Tables S1 to S7.

ASM does not own the copyrights to Supplemental Material that may be linked to, or accessed through, an article. The authors have granted ASM a non-exclusive, world-wide license to publish the Supplemental Material files. Please contact the corresponding author directly for reuse.
