# Supplementary figures and images for: Gut microbiota dynamics in SAMP8 mice: insights from machine learning and longitudinal analysis
Source: Microbiol Spectr. 2025 Sep 23;13(11):e00635-25. doi: 10.1128/spectrum.00635-25 (PMC12584719; doi:10.1128/spectrum.00635-25)

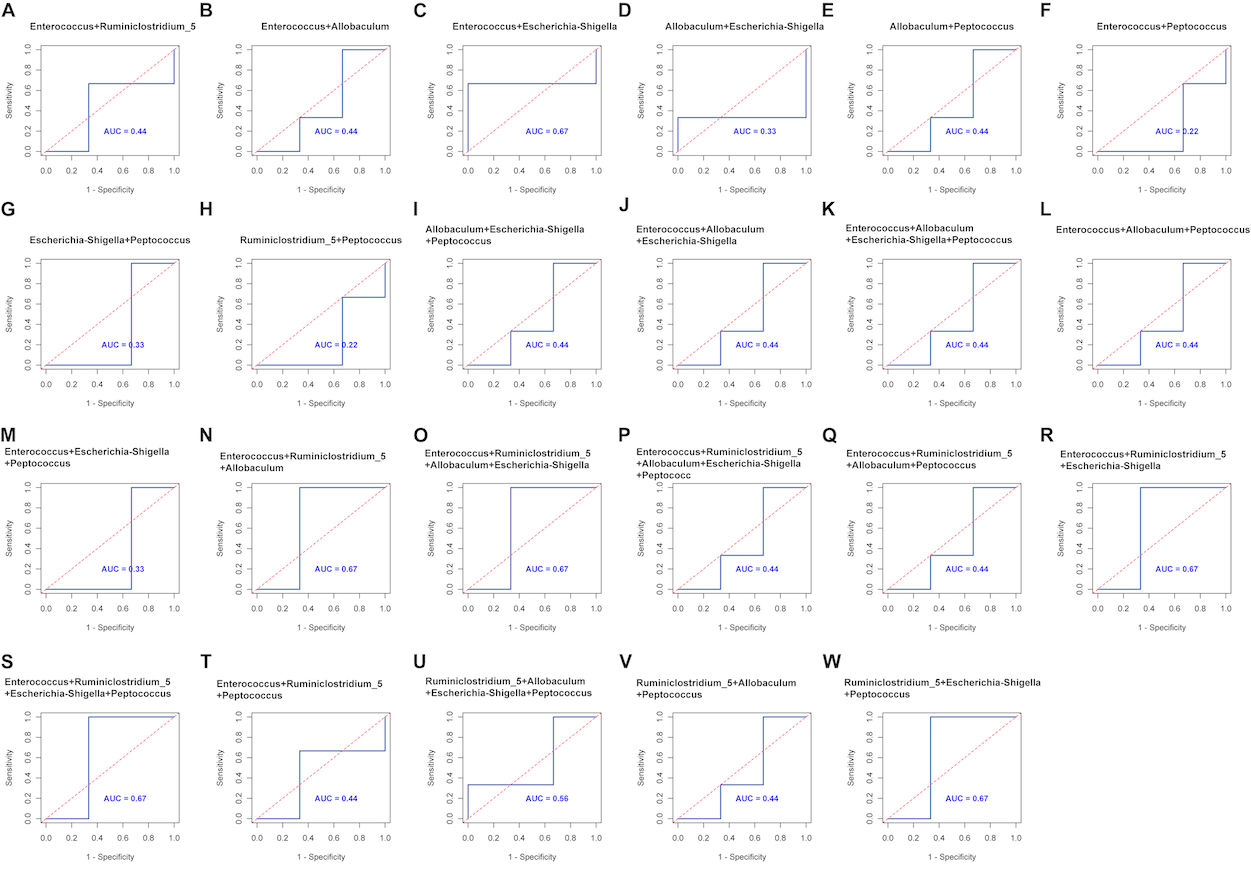

Supplement: Fig. S1 — ROC curve analysis for various combinations of the top five genera identified by the Random Forest model. [file spectrum.00635-25-s0001.tif]
