# Supplementary material for: Gut microbiota dynamics in SAMP8 mice: insights from machine learning and longitudinal analysis
Source: Microbiol Spectr. 2025 Sep 23;13(11):e00635-25. doi: 10.1128/spectrum.00635-25 (PMC12584719; doi:10.1128/spectrum.00635-25)
Supplement: Supplemental tables — Tables S1 to S7. [file spectrum.00635-25-s0002.docx]

Supplemental Materials

**1 Table S1 Kruskal-Wallis test for phylum**

| Phylum | Chi_Squared | P_Value |
| --- | --- | --- |
| *Actinobacteria* | 10.1221351900203 | 0.0175559683106586 |
| *Bacteroidetes* | 7.98 | 0.0464268588265063 |
| *Cyanobacteria* | 2.48382651581085 | 0.478221086368964 |
| *Deferribacteres* | 10.7363346382485 | 0.0132404920708995 |
| *Epsilonbacteraeota* | 1.88666666666667 | 0.596259254374615 |
| *Firmicutes* | 12.0333333333333 | 0.0072698424895873 |
| *Patescibacteria* | 9.56666666666666 | 0.022632613630289 |
| *Proteobacteria* | 8.65333333333332 | 0.0342733898983387 |
| *Tenericutes* | 11.32 | 0.0101155808749192 |
| *Verrucomicrobia* | 18.9383273164862 | 0.000281544819794453 |

**2 Table S2 Kruskal-Wallis test for F/B ratio**

|  | Group1 | Group2 | p | p.adj | p.adj.significance |
| --- | --- | --- | --- | --- | --- |
| Log2_F/B | 1m | 3m | 0.394 | 1 | ns |
| Log2_F/B | 1m | 7m | 0.093 | 0.559 | ns |
| Log2_F/B | 1m | 10m | 0.002 | 0.013 | * |
| Log2_F/B | 3m | 7m | 0.093 | 0.559 | ns |
| Log2_F/B | 3m | 10m | 0.009 | 0.052 | ns |
| Log2_F/B | 7m | 10m | 0.31 | 1 | ns |

**3 Table S3 Kruskal-Wallis tests for genera**

| Genus | Chi_Squared | P_Value |
| --- | --- | --- |
| *norank_f__Muribaculaceae* | 7.433333333 | 0.059296375 |
| *Alistipes* | 7.246666667 | 0.064437709 |
| *Lachnospiraceae_NK4A136_group* | 7.5 | 0.057558452 |
| *Bacteroides* | 8.753333333 | 0.032756751 |
| *Alloprevotella* | 9.153333333 | 0.027320196 |
| *Odoribacter* | 2.579454835 | 0.461102769 |
| *unclassified_f__Lachnospiraceae* | 10.96666667 | 0.011907494 |
| *Allobaculum* | 16.01362911 | 0.001126712 |
| *Prevotellaceae_UCG.001* | 7.18 | 0.066376565 |
| *Helicobacter* | 1.886666667 | 0.596259254 |

**4 Table S4 Classification of microbial genera into clusters based on longitudinal analysis**

| Genus | Cluster |
| --- | --- |
| *[Eubacterium]_fissicatena_group* | 1 |
| *Acetatifactor* | 1 |
| *Alistipes* | 1 |
| *Anaerococcus* | 1 |
| *Anaerotruncus* | 1 |
| *Atopostipes* | 1 |
| *Butyricicoccus* | 1 |
| *Candidatus_Arthromitus* | 1 |
| *Candidatus_Saccharimonas* | 1 |
| *Caulobacter* | 1 |
| *Clostridium_sensu_stricto_1* | 1 |
| *Enterococcus* | 1 |
| *Erysipelatoclostridium* | 1 |
| *Escherichia-Shigella* | 1 |
| *GCA.900066225* | 1 |
| *Intestinimonas* | 1 |
| *Lachnospiraceae_FCS020_group* | 1 |
| *Lachnospiraceae_UCG-004* | 1 |
| *Mucispirillum* | 1 |
| *norank_f__Erysipelotrichaceae* | 1 |
| *norank_f__norank_o__Mollicutes_RF39* | 1 |
| *norank_f__Peptococcaceae* | 1 |
| *norank_f__Ruminococcaceae* | 1 |
| *Oscillibacter* | 1 |
| *Peptococcus* | 1 |
| *Rikenella* | 1 |
| *Ruminiclostridium* | 1 |
| *Ruminococcaceae_UCG-009* | 1 |
| *Ruminococcaceae_UCG-010* | 1 |
| *Ruminococcaceae_UCG-014* | 1 |
| *Tyzzerella_3* | 1 |
| *UBA1819* | 1 |
| *unclassified_f__Erysipelotrichaceae* | 1 |
| *unclassified_f__Lachnospiraceae* | 1 |
| *unclassified_f__Rikenellaceae* | 1 |
| *unclassified_f__Ruminococcaceae* | 1 |
| *unclassified_p__Firmicutes* | 1 |
| *[Eubacterium]_coprostanoligenes_group* | 2 |
| *Akkermansia* | 2 |
| *Ammoniibacillus* | 2 |
| *Anaerovorax* | 2 |
| *Bilophila* | 2 |
| *Christensenellaceae_R.7_group* | 2 |
| *Desulfovibrio* | 2 |
| *Enterorhabdus* | 2 |
| *Family_XIII_AD3011_group* | 2 |
| *Family_XIII_UCG-001* | 2 |
| *Lactobacillus* | 2 |
| *norank_f__Christensenellaceae* | 2 |
| *norank_f__Desulfovibrionaceae* | 2 |
| *norank_f__Muribaculaceae* | 2 |
| *norank_f__norank_o__Gastranaerophilales* | 2 |
| *Novibacillus* | 2 |
| *Parabacteroides* | 2 |
| *Parasutterella* | 2 |
| *Ruminiclostridium_5* | 2 |
| *Ruminiclostridium_9* | 2 |
| *Ruminococcaceae_NK4A214_group* | 2 |
| *Ruminococcaceae_UCG-013* | 2 |
| *Ruminococcus_1* | 2 |
| *Thermobifida* | 2 |
| *Turicibacter* | 2 |
| *unclassified_f__Prevotellaceae* | 2 |
| *[Eubacterium]_nodatum_group* | 3 |
| *[Eubacterium]_xylanophilum_group* | 3 |
| *Alloprevotella* | 3 |
| *Anaeroplasma* | 3 |
| *Azospirillum* | 3 |
| *Bacteroides* | 3 |
| *Blautia* | 3 |
| *GCA.900066575* | 3 |
| *Halomonas* | 3 |
| *Harryflintia* | 3 |
| *Helicobacter* | 3 |
| *Lachnospiraceae_NK4A136_group* | 3 |
| *Lachnospiraceae_UCG-006* | 3 |
| *Lactococcus* | 3 |
| *Marvinbryantia* | 3 |
| *Methylobacterium* | 3 |
| *norank_f__Clostridiales_vadinBB60_group* | 3 |
| *norank_f__Lachnospiraceae* | 3 |
| *Odoribacter* | 3 |
| *Prevotellaceae_UCG-001* | 3 |
| *Proteus* | 3 |
| *Pseudomonas* | 3 |
| *Rikenellaceae_RC9_gut_group* | 3 |
| *Rodentibacter* | 3 |
| *Shewanella* | 3 |
| *Tyzzerella* | 3 |
| *unclassified_k__norank_d__Bacteria* | 3 |
| *[Eubacterium]_brachy_group* | 4 |
| *[Eubacterium]_ventriosum_group* | 4 |
| *A2* | 4 |
| *Adlercreutzia* | 4 |
| *Allobaculum* | 4 |
| *ASF356* | 4 |
| *Bacillus* | 4 |
| *Bifidobacterium* | 4 |
| *Candidatus_Stoquefichus* | 4 |
| *Cerasibacillus* | 4 |
| *Corynebacterium_1* | 4 |
| *Dubosiella* | 4 |
| *Faecalibaculum* | 4 |
| *Gallicola* | 4 |
| *Janibacter* | 4 |
| *Jeotgalicoccus* | 4 |
| *Lachnoclostridium* | 4 |
| *Lachnospiraceae_UCG-001* | 4 |
| *Lachnospiraceae_UCG-010* | 4 |
| *Muribaculum* | 4 |
| *norank_f__norank_o__Rhodospirillales* | 4 |
| *Parvibacter* | 4 |
| *Prevotellaceae_NK3B31_group* | 4 |
| *Pseudogracilibacillus* | 4 |
| *Romboutsia* | 4 |
| *Roseburia* | 4 |
| *Ruminiclostridium_6* | 4 |
| *Ruminococcaceae_UCG-005* | 4 |
| *Sporosarcina* | 4 |
| *Staphylococcus* | 4 |
| *Streptococcus* | 4 |
| *unclassified_f__Burkholderiaceae* | 4 |
| *unclassified_f__Enterobacteriaceae* | 4 |
| *unclassified_o__Bacteroidales* | 4 |
| *unclassified_o__Clostridiales* | 4 |
| *unclassified_o__Coriobacteriales* | 4 |
| *Ureibacillus* | 4 |
| *Yaniella* | 4 |

**5Table S5 Linear regression results for 128 genera**

| Genus | Coefficient | P_value |
| --- | --- | --- |
| *[Eubacterium]_brachy_group* | -0.0921002259195759 | 0.628732119979926 |
| *[Eubacterium]_coprostanoligenes_group* | 0.218407597128306 | 0.119570828882522 |
| *[Eubacterium]_fissicatena_group* | -0.121825468170758 | 0.508905837650104 |
| *[Eubacterium]_nodatum_group* | 0.102046292211113 | 0.588638244967909 |
| *[Eubacterium]_ventriosum_group* | 0.00837501142989301 | 0.966239249603628 |
| *[Eubacterium]_xylanophilum_group* | -0.0512753597163134 | 0.793302416915179 |
| *A2* | -0.110135277609067 | 0.556030502367643 |
| *Acetatifactor* | -0.174962484823567 | 0.294703675534815 |
| *Adlercreutzia* | -0.0781514999268534 | 0.684961232086786 |
| *Akkermansia* | 0.198470717082642 | 0.19993896169505 |
| *Alistipes* | -0.228996117586769 | 0.0768871383278914 |
| *Allobaculum* | -0.167966938636288 | 0.322903623760801 |
| *Alloprevotella* | 0.201460784277606 | 0.187885615489784 |
| *Ammoniibacillus* | 0.239378928924494 | 0.0350326877495466^*^ |
| *Anaerococcus* | -0.236171837506598 | 0.0479608865662608^*^ |
| *Anaeroplasma* | 0.155021178447209 | 0.375089651456808 |
| *Anaerotruncus* | -0.213558530396898 | 0.139118041796181 |
| *Anaerovorax* | 0.138835838966257 | 0.440334840776381 |
| *ASF356* | -0.171774043581172 | 0.307556693090563 |
| *Atopostipes* | -0.20750553505885 | 0.163518446078476 |
| *Azospirillum* | 0.0717948717948718 | 0.710585619291847 |
| *Bacillus* | -0.204344762173621 | 0.176259928920752 |
| *Bacteroides* | 0.0799598296671288 | 0.677671621856779 |
| *Bifidobacterium* | -0.188785630218777 | 0.238980795000619 |
| *Bilophila* | 0.0714388676365002 | 0.712020717933976 |
| *Blautia* | -0.0969871595211457 | 0.609032260832588 |
| *Butyricicoccus* | -0.18669409523507 | 0.247412042064734 |
| *Candidatus_Arthromitus* | -0.170707643059059 | 0.311855491026659 |
| *Candidatus_Saccharimonas* | -0.105752435041658 | 0.573698305591978 |
| *Candidatus_Stoquefichus* | -0.147943204457525 | 0.403621876777103 |
| *Caulobacter* | -0.174358974358974 | 0.297136503994485 |
| *Cerasibacillus* | -0.182534318506323 | 0.264180638146 |
| *Christensenellaceae_R.7_group* | 0.161465902371829 | 0.349110138758354 |
| *Clostridium_sensu_stricto_1* | -0.16907046577787 | 0.318455163636963 |
| *Corynebacterium_1* | -0.14726067143981 | 0.406373255332373 |
| *Desulfovibrio* | 0.082696256871918 | 0.666640731139546 |
| *Dubosiella* | -0.153142982081161 | 0.382660903059305 |
| *Enterococcus* | -0.183823633712672 | 0.258983242389807 |
| *Enterorhabdus* | 0.179350070915458 | 0.277016750551975 |
| *Erysipelatoclostridium* | -0.223695577652797 | 0.0982542979043088 |
| *Escherichia-Shigella* | -0.240306994897091 | 0.0312915342102944^*^ |
| *Faecalibaculum* | -0.117790345806678 | 0.525171935922674 |
| *Family_XIII_AD3011_group* | 0.235900894585292 | 0.0490530923995863^*^ |
| *Family_XIII_UCG-001* | 0.226265736859682 | 0.0878936549142726 |
| *Gallicola* | -0.131268706195424 | 0.470838928183408 |
| *GCA.900066225* | -0.0418025037948195 | 0.831488719940968 |
| *GCA.900066575* | 0.0505670621016198 | 0.796157655881261 |
| *Halomonas* | -0.08898761088128 | 0.641279472334912 |
| *Harryflintia* | -0.00262961553706368 | 0.989399680880631 |
| *Helicobacter* | -0.144526283511252 | 0.417395925464308 |
| *Intestinimonas* | -0.23301731770022 | 0.0606771624418298 |
| *Janibacter* | -0.0923076923076923 | 0.627895796232375 |
| *Jeotgalicoccus* | -0.169471309370823 | 0.316839311455391 |
| *Lachnoclostridium* | -0.180922800716624 | 0.27067687403926 |
| *Lachnospiraceae_FCS020_group* | -0.219096902997024 | 0.116792148092012 |
| *Lachnospiraceae_NK4A136_group* | -0.0880516913859016 | 0.645052284436611 |
| *Lachnospiraceae_UCG-001* | -0.211908062408944 | 0.145771290958279 |
| *Lachnospiraceae_UCG-004* | -0.246695574091521 | 0.0055383481548393^*^ |
| *Lachnospiraceae_UCG-006* | -0.00206181961461976 | 0.991688539418219 |
| *Lachnospiraceae_UCG-010* | -0.229093532132697 | 0.0764944477390423 |
| *Lactobacillus* | 0.231960679293772 | 0.0649366080315956 |
| *Lactococcus* | 0.166677773257035 | 0.328100415544661 |
| *Marvinbryantia* | 0.0645473444837575 | 0.739801335901865 |
| *Methylobacterium* | -0.0870359051233631 | 0.649147049769494 |
| *Mucispirillum* | -0.218210596668158 | 0.120364963125647 |
| *Muribaculum* | -0.116972487414246 | 0.528468828503371 |
| *norank_f__Christensenellaceae* | 0.110082481055284 | 0.556243332080054 |
| *norank_f__Clostridiales_vadinBB60_group* | -0.101015947823653 | 0.592791695978517 |
| *norank_f__Desulfovibrionaceae* | 0.184563187067481 | 0.256002007507263 |
| *norank_f__Erysipelotrichaceae* | -0.128369248393723 | 0.482527016247224 |
| *norank_f__Lachnospiraceae* | -0.0734078532037999 | 0.704083483360851 |
| *norank_f__Muribaculaceae* | 0.0692373213759532 | 0.720895434632642 |
| *norank_f__norank_o__Gastranaerophilales* | -0.013442143377801 | 0.945812987699292 |
| *norank_f__norank_o__Mollicutes_RF39* | -0.21379637606666 | 0.138159255249208 |
| *norank_f__norank_o__Rhodospirillales* | -0.0235733711909914 | 0.904972702730956 |
| *norank_f__Peptococcaceae* | -0.241780197 | 0.025352865^*^ |
| *norank_f__Ruminococcaceae* | -0.182193785415491 | 0.265553370921056 |
| *Novibacillus* | 0.235446085003943 | 0.0508864884351993 |
| *Odoribacter* | 0.118004275093288 | 0.524309559398393 |
| *Oscillibacter* | -0.181733845361451 | 0.267407448553274 |
| *Parabacteroides* | 0.218219214394041 | 0.12033022396201 |
| *Parasutterella* | 0.202984757530237 | 0.181742282907671 |
| *Parvibacter* | -0.109341982011208 | 0.559228379157907 |
| *Peptococcus* | -0.245396197886956 | 0.0107763010914414^*^ |
| *Prevotellaceae_NK3B31_group* | -0.117934318236934 | 0.524591564501986 |
| *Prevotellaceae_UCG-001* | 0.0675925793213054 | 0.72752560181966 |
| *Proteus* | 0.0479831816117765 | 0.806573611131219 |
| *Pseudogracilibacillus* | -0.224149339343906 | 0.0964251260492963 |
| *Pseudomonas* | 0.176659087828664 | 0.287864450173002 |
| *Rikenella* | -0.218575081769624 | 0.11889567670893 |
| *Rikenellaceae_RC9_gut_group* | 0.0360713307243492 | 0.854591817188088 |
| *Rodentibacter* | 0.077292584130499 | 0.688423632353934 |
| *Romboutsia* | -0.112049840634251 | 0.548312652075447 |
| *Roseburia* | -0.202028850132558 | 0.185595668819467 |
| *Ruminiclostridium* | -0.168363087173553 | 0.321306697953778 |
| *Ruminiclostridium_5* | 0.206044814566329 | 0.169406798632939 |
| *Ruminiclostridium_6* | -0.186306984734048 | 0.248972534382892 |
| *Ruminiclostridium_9* | 0.00922820624921204 | 0.962799911332197 |
| *Ruminococcaceae_NK4A214_group* | 0.0494767252803737 | 0.800552944123934 |
| *Ruminococcaceae_UCG-005* | -0.209470423545071 | 0.155597727367187 |
| *Ruminococcaceae_UCG-009* | -0.194040115634787 | 0.217799287121345 |
| *Ruminococcaceae_UCG-010* | -0.0635309384003822 | 0.743898599812421 |
| *Ruminococcaceae_UCG-013* | 0.176285769361109 | 0.289369345026813 |
| *Ruminococcaceae_UCG-014* | -0.174564942275918 | 0.296306220777344 |
| *Ruminococcus_1* | 0.21320033026331 | 0.140561992697411 |
| *Shewanella* | -0.0981677154207215 | 0.604273287858261 |
| *Sporosarcina* | -0.185297732129594 | 0.253040961698016 |
| *Staphylococcus* | -0.146608768871371 | 0.409001158599137 |
| *Streptococcus* | -0.0784492999410951 | 0.683760761850655 |
| *Thermobifida* | 0.141912837425141 | 0.427931063443061 |
| *Turicibacter* | 0.114933057506813 | 0.536690033290026 |
| *Tyzzerella* | 0.0165992801070489 | 0.933086162670384 |
| *Tyzzerella_3* | -0.201934701489005 | 0.185975194134956 |
| *UBA1819* | -0.0205793376723799 | 0.917042037698001 |
| *unclassified_f__Burkholderiaceae* | -0.0923076923076923 | 0.627895796232375 |
| *unclassified_f__Enterobacteriaceae* | -0.216476174631208 | 0.127356641878755 |
| *unclassified_f__Erysipelotrichaceae* | -0.226020490102643 | 0.0888822761978667 |
| *unclassified_f__Lachnospiraceae* | -0.234676630100804 | 0.0539882603125993 |
| *unclassified_f__Prevotellaceae* | 0.223515525708818 | 0.0989801104945301 |
| *unclassified_f__Rikenellaceae* | -0.178746628043352 | 0.279449306537624 |
| *unclassified_f__Ruminococcaceae* | -0.145059470386171 | 0.415246580457504 |
| *unclassified_k__norank_d__Bacteria* | 0.126437592561162 | 0.490313769848778 |
| *unclassified_o__Bacteroidales* | 0.0429532140579425 | 0.826850058573513 |
| *unclassified_o__Clostridiales* | -0.115098022384545 | 0.536025038608182 |
| *unclassified_o__Coriobacteriales* | -0.198269928867111 | 0.200748364868267 |
| *unclassified_p__Firmicutes* | -0.0193533109351177 | 0.921984309479058 |
| *Ureibacillus* | -0.221209832367556 | 0.108274657497425 |
| *Yaniella* | -0.211869377696485 | 0.145927234019663 |

**6 Table S6 LefSE analysis results**

| Taxa | Group | LDA | p.unadj | p.adj | Significance |
| --- | --- | --- | --- | --- | --- |
| *Allobaculum* | Y | 4.267315867 | 0.0000864 | 0.002211243 | ** |
| *unclassified_f__Lachnospiraceae* | Y | 3.957780197 | 0.001496164 | 0.00832648 | ** |
| *Akkermansia* | O | 3.949539231 | 0.0000229 | 0.001015981 | ** |
| *norank_f__norank_o__Mollicutes_RF39* | Y | 3.67573369 | 0.000174318 | 0.003187528 | ** |
| *Parabacteroides* | O | 3.497373131 | 0.000344158 | 0.004004746 | ** |
| *Mucispirillum* | Y | 3.346991326 | 0.001492607 | 0.00832648 | ** |
| *Rikenella* | Y | 3.301770245 | 0.000275504 | 0.003918276 | ** |
| *Enterococcus* | Y | 3.294417168 | 0.000340076 | 0.004004746 | ** |
| *Ruminiclostridium_5* | O | 3.237743999 | 0.000809985 | 0.005183907 | ** |
| *Dubosiella* | Y | 3.156453092 | 0.000532006 | 0.004005689 | ** |
| *unclassified_f__Prevotellaceae* | O | 3.143093118 | 0.000428568 | 0.004005689 | ** |
| *Anaeroplasma* | O | 3.012692732 | 0.0000317 | 0.001015981 | ** |
| *[Eubacterium]_coprostanoligenes_group* | O | 2.974509958 | 0.00052755 | 0.004005689 | ** |
| *Erysipelatoclostridium* | Y | 2.712309812 | 0.000526071 | 0.004005689 | ** |
| *Ruminiclostridium_6* | Y | 2.512304554 | 0.000801497 | 0.005183907 | ** |
| *Escherichia-Shigella* | Y | 2.50782323 | 0.000524595 | 0.004005689 | ** |
| *Bifidobacterium* | Y | 2.392035861 | 0.0000314 | 0.001015981 | ** |
| *Lactococcus* | O | 2.14765382 | 0.0000117 | 0.001015981 | ** |
| *Novibacillus* | O | 2.129742216 | 0.000216624 | 0.003465988 | ** |
| *Peptococcus* | Y | 2.061863073 | 0.00177288 | 0.009455361 | ** |
| *Cerasibacillus* | Y | 1.986747725 | 0.000115032 | 0.002454011 | ** |
| *unclassified_f__Erysipelotrichaceae* | Y | 1.954789989 | 0.000745778 | 0.005183907 | ** |
| *Family_XIII_UCG-001* | O | 1.929228197 | 0.000483033 | 0.004005689 | ** |
| *Ureibacillus* | Y | 1.894302375 | 0.001281349 | 0.007810128 | ** |

**7 Table S7 Random Forest analysis results**

| Genus | Overall | Cumulative_importance |
| --- | --- | --- |
| *Enterococcus* | 100 | 0.133835682982578 |
| *Ruminiclostridium_5* | 80.3090395632613 | 0.241317834578817 |
| *Allobaculum* | 65.6303106381887 | 0.329154609065024 |
| *Escherichia-Shigella* | 52.8887717324018 | 0.399938657934181 |
| *Peptococcus* | 52.3657320042617 | 0.470022693010911 |
| *Bifidobacterium* | 47.6753742822031 | 0.533829355795997 |
| *Parabacteroides* | 47.0892248504553 | 0.596851541485806 |
| *Akkermansia* | 39.3955612887961 | 0.649576860001486 |
| *Dubosiella* | 37.6758440897015 | 0.70000058325839 |
| *[Eubacterium]_coprostanoligenes_group* | 28.1558782014879 | 0.737683195149094 |
| *Lactococcus* | 27.5015792045683 | 0.774490121508522 |
| *Ruminiclostridium_6* | 26.32481013272 | 0.809722110943515 |
| *Rikenella* | 22.287522240596 | 0.839550768554111 |
| *Anaeroplasma* | 19.6313933715242 | 0.865824577951886 |
| *norank_f__norank_o__Mollicutes_RF39* | 18.2504398450693 | 0.890250178765859 |
| *Cerasibacillus* | 17.2006189161645 | 0.913270744569539 |
| *Erysipelatoclostridium* | 16.7164000191786 | 0.935643252705306 |
| *Mucispirillum* | 14.2958635253537 | 0.95477621929272 |
| *unclassified_f__Lachnospiraceae* | 12.6208768451995 | 0.971667456016883 |
| *unclassified_f__Prevotellaceae* | 10.402790089167 | 0.985590101181964 |
| *Family_XIII_UCG-001* | 7.43285832813481 | 0.99553791789055 |
| *unclassified_f__Erysipelotrichaceae* | 1.75128462063012 | 0.99788176162354 |
| *Ureibacillus* | 1.58271570724245 | 1 |
| *Novibacillus* | 0 | 1 |

**6 Fig. S1**

ROC curve analysis for various combinations of the top five genera identified by the Random Forest model. (A) *Enterococcus*+*Ruminiclostridium_5*; (B) *Enterococcus*+*Allobaculum*; (C) *Enterococcus*+*Escherichia-Shigella*; (D) *Allobaculum*+*Escherichia-Shigella*; (E) *Allobaculum*+*Peptococcus*; (F) *Enterococcus*+*Peptococcus*; (G) *Escherichia-Shigella*+*Peptococcus*; (H) *Ruminiclostridium_5*+*Peptococcus*; (I) *Allobaculum*+*Escherichia-Shigella*+*Peptococcus*; (J) *Enterococcus*+*Allobaculum*+*Escherichia-Shigella*; (K) *Enterococcus*+*Allobaculum*+*Escherichia-Shigella*+*Peptococcus*; (L) *Enterococcus*+*Allobaculum*+*Peptococcus*; (M) *Enterococcus*+*Escherichia-Shigella*+*Peptococcus*; (N) *Enterococcus*+*Ruminiclostridium_5*+*Allobaculum*; (O) *Enterococcus*+*Ruminiclostridium_5*+*Allobaculum*+*Escherichia-Shigella*; (P) *Enterococcus*+*Ruminiclostridium_5*+*Allobaculum*+*Escherichia-Shigella*+*Peptococcus*; (Q) *Enterococcus*+*Ruminiclostridium_5*+*Allobaculum*+*Peptococcus*; (R) *Enterococcus*+*Ruminiclostridium_5*+*Escherichia-Shigella*; (S) Enterococcus+Ruminiclostridium_5

+*Escherichia-Shigella*+*Peptococcus*; (T) *Enterococcus*+*Ruminiclostridium_5*+*Peptococcus;* (U) *Ruminiclostridium_5*+*Allobaculum*+*Escherichia-Shigella*+*Peptococcus*; (V) *Ruminiclostridium_5*

+*Allobaculum*+*Peptococcus*; (W) *Ruminiclostridium_5*+*Escherichia-Shigella*+*Peptococcus*.
